# Volatile Compound-Mediated Recognition and Inhibition Between *Trichoderma* Biocontrol Agents and *Fusarium oxysporum*

**DOI:** 10.3389/fmicb.2018.02614

**Published:** 2018-10-31

**Authors:** Ningxiao Li, Alsayed Alfiky, Wenzhao Wang, Md Islam, Khoshnood Nourollahi, Xingzhong Liu, Seogchan Kang

**Affiliations:** ^1^Intercollege Graduate Degree Program in Plant Biology, Pennsylvania State University, University Park, PA, United States; ^2^Department of Plant Pathology and Environmental Microbiology, Pennsylvania State University, University Park, PA, United States; ^3^Genetics Department, Faculty of Agriculture, Tanta University, Tanta, Egypt; ^4^State Key Laboratory of Mycology, Institute of Microbiology, Chinese Academy of Sciences, Beijing, China; ^5^Plant Protection Department, Ilam University, Ilam, Iran

**Keywords:** antifungal metabolites, biological control, fungal chemical ecology, *Fusarium oxysporum*, *Trichoderma*, volatile compounds

## Abstract

Certain *Trichoderma* strains protect plants from diverse pathogens using multiple mechanisms. We report a novel mechanism that may potentially play an important role in *Trichoderma*-based biocontrol. *Trichoderma virens* and *T. viride* significantly increased the amount/activity of secreted antifungal metabolites in response to volatile compounds (VCs) produced by 13 strains of *Fusarium oxysporum*, a soilborne fungus that infects diverse plants. This response suggests that both *Trichoderma* spp. recognize the presence of *F. oxysporum* by sensing pathogen VCs and prepare for attacking pathogens. However, *T. asperellum* did not respond to any, while *T. harzianum* responded to VCs from only a few strains. Gene expression analysis via qPCR showed up-regulation of several biocontrol-associated genes in *T. virens* in response to *F. oxysporum* VCs. Analysis of VCs from seven *F. oxysporum* strains tentatively identified a total of 28 compounds, including six that were produced by all of them. All four *Trichoderma* species produced VCs that inhibited *F. oxysporum* growth. Analysis of VCs produced by *T. virens* and *T. harzianum* revealed the production of compounds that had been reported to display antifungal activity. *F. oxysporum* also recognizes *Trichoderma* spp. by sensing their VCs and releases VCs that inhibit *Trichoderma*, suggesting that both types of VC-mediated interaction are common among fungi.

## Introduction

The global need for quality food, feed, and fiber will continue to increase. Efforts to meet this need face multiple complex challenges, one of which is how to protect crop plants from diverse diseases and pests without heavily relying on synthetic pesticides. Due to the multi-faceted negative impact of synthetic pesticides, we cannot afford to keep expanding their use. Biocontrol agents (BCAs) have long been touted as an environment-friendly alternative to synthetic pesticides. However, the following hurdles hinder the full realization of their potential: (a) inconsistent efficacy under different production systems and environmental conditions; (b) limited knowledge about underlying reasons for biocontrol failures; and (c) poor understanding of how BCAs work in light of complex and varied biotic and abiotic factors (Mazzola and Freilich, 2016; Timmusk et al., 2017). Until we convincingly demonstrate that biocontrol can work reliably and effectively, biocontrol will continue to be perceived by risk-averse growers as a risky and high-cost practice.

Several *Trichoderma* species have been marketed as bio-fungicides against diverse crop pathogens or as bio-fertilizers (Woo et al., 2014). *Trichoderma* BCAs have been shown to control pathogens via multiple synergistic mechanisms, including mycoparasitism, antibiosis, competition for space and nutrients, and induction of plant systemic resistance (Howell, 2003; Harman et al., 2004; Harman, 2006; Shoresh et al., 2010; Sawant, 2014; Woo et al., 2014). Compared to these mechanisms, relatively little is known about how *Trichoderma* BCAs recognize pathogens. The prevailing model is that bioactive molecules diffused from pathogens activate the biocontrol machinery (Druzhinina et al., 2011). Considering that water may not be readily available to mediate this form of recognition in the soil, waterborne molecules may not be the only signals. In this study, we investigated if and how volatile compounds (VCs) from both the *Trichoderma* and pathogen sides affect their interaction. Since previous studies already showed strong inhibition of plant pathogenic fungi such as *Fusarium oxysporum, Rhizoctonia solani, Sclerotium rolfsii, Sclerotinia sclerotiorum*, and *Alternaria brassicicola* by *Trichoderma* VCs (Amin et al., 2010; Meena et al., 2017), we focused on investigating how pathogen VCs affect *Trichoderma* using *F. oxysporum*, a soilborne fungal species complex consisting of genetically and phenotypically diverse members (O'Donnell et al., 2009; Kang et al., 2014). Pathogenic *F. oxysporum* strains collectively infect many economically important plants, causing vascular wilt, root rot, or damping-off diseases (Nelson, 1981; Beckman, 1987; Gordon and Martyn, 1997; Sharma and Muehlbauer, 2007).

Due to their ability to move through the air, VCs perform many crucial functions in animals and plants. Many olfactory receptors (~350 in humans and ~1,000 in mice) are produced by animals (Buck, 2004) because their survival heavily depends on the ability to track foods, find mates, and recognize threats via volatile cues. Plant VCs function as homing cues to pollinators, seed dispersers, and parasitoids (Baldwin, 2010; Herrmann, 2010), mediate communication within and between plants (Baldwin, 2010), and protect plants from diverse predators (Baldwin, 2010; Huang et al., 2012; Arasimowicz-Jelonek and Floryszak-Wieczorek, 2014). Similarly, microbial VCs appear to participate in microbial interactions (Bailly and Weisskopf, 2012; Bennett et al., 2012; Bitas et al., 2013) and also affect plant growth, development, and stress resistance (Bailly and Weisskopf, 2012; Bitas et al., 2013; Li et al., 2016; Li and Kang, 2018). VCs produced by biocontrol agent *Paenibacillus polymyxa* and soilborne fungal pathogen *Verticillium longisporum* appeared to affect the production of antimicrobial VCs and other metabolites in the other side (Rybakova et al., 2017). VCs from *Aspergillus flavus* and *Ralstonia solanacearum*, soilborne fungal and bacterial pathogens, respectively, affected multiple traits of the other side (Spraker et al., 2014). Here, we report that besides antibiosis, some VCs from both *Trichoderma* and *F. oxysporum* likely function as signals in their interactions.

## Materials and methods

### Fungal cultures and growth conditions

The following *Trichoderma* strains were cultured from commercial biocontrol products: (a) *T. virens* G-41 from RootShield® Plus^+^ (BioWorks, Victor, NY, United States), (b) *T. harzianum* T-22 from RootShield® (BioWorks), (c) *T. asperellum* ICC 012 from BIO-TAM® 2.0 (Isagro, Morrisville, NC, United States), and (d) *T. harzianum* and *T. viride* from Custom GP (Custom Biologicals, Boca Raton, FL, United States). Genomic DNA from these strains was extracted as previously described (Cassago et al., 2002) to confirm identity by sequencing the ITS regions. Table 1 lists the *F. oxysporum* strains used. Fungal strains, preserved as spore suspension in 20% glycerol at −80°C, were activated by inoculating on 0.5x potato dextrose agar (PDA; Difco, Houston, TX). For all the experiments described below, a plug of culture (4 mm in diameter), cut from the actively growing margin of stock culture using a cork borer, was placed on 15 mL 0.75x PDA. We only used one incubator for each experiment to avoid potential variation caused by using multiple incubators. We randomly placed VC-treated and control plates in incubator shelves.

**Table 1:** *Fusarium oxysporum* strains used.

**Accession #^a^**	***forma specialis* (f.sp.)**	**Geographical origin^b^**
NRRL26379	*radicis-lycopersici*	Florida, USA
NRRL38499	*lycopersici*	N/A
NRRL54003	*lycopersici*	USA
9605	*ciceris*	Tunisia
W6-1	*ciceris*	California, USA
NRRL38272	*conglutinans*	Australia
NRRL38487	*conglutinans*	N/A
NRRL37611	*pisi*	Australia
NRRL37616	*pisi*	N/A
NRRL26029	*cubense*	N/A
NRRL36118	*cubense*	N/A
NRRL22518	*melonis*	N/A
NRRL22519	*melonis*	N/A

a *NRRL corresponds to the ARS Culture Collection at the National Center for Agricultural Utilization Research. Maria Jimenez-Gasco at Penn State provided two strains of f. sp. ciceris*.

b *N/A, not available*.

### Evaluation of VC-mediated inhibition between *F. oxysporum* and *Trichoderma*

**Figure 1:**
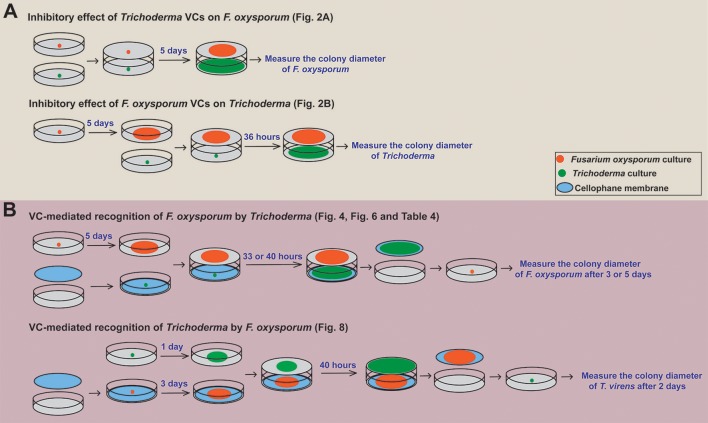
Overview of the experimental setups employed. A schematic representation of how we determined **(A)** the inhibitory effect of *Trichoderma* VCs on the growth of *F. oxysporum* and vice versa and **(B)** if *Trichoderma* and *F. oxysporum* sense VCs from the other side to prepare for subsequent attack is shown. The duration of VC exposure varied significantly among the experiments because *Trichoderma* grew much faster than *F. oxysporum*. Specific figures and table generated using each method are noted.

Sandwiched Petri plates, a setup described in Dennis and Webster (1971), was employed to determine if and how *Trichoderma* VCs affect the growth of *F. oxysporum*. After inoculating *F. oxysporum* and *Trichoderma* on PDA plate, *F. oxysporum* plate was placed on top of *Trichoderma* plate, sealed with three layers of Parafilm, and incubated at 25°C (Figure 1A). Each plate of *F. oxysporum* also was sandwiched with an un-inoculated PDA plate (control treatment). Colony diameter of *F. oxysporum* was measured 5 days later. We evaluated the inhibitory effect of *F. oxysporum* VCs on *Trichoderma* in the same way except that 5-day-old (after the inoculation of culture plug) *F. oxysporum* culture was used to ensure enough *F. oxysporum* biomass. Colony diameter of *Trichoderma* was measured 36 h later. The duration of VC exposure between the two experiments was different because *Trichoderma* grew much faster than *F. oxysporum*. Each treatment included three biological replicates and was repeated three times.

### VC-mediated recognition of *F. oxysporum* by *Trichoderma*

To determine whether *Trichoderma* recognizes *F. oxysporum* VCs and prepares for attack, we compared the activity of antifungal molecules secreted by *Trichoderma* in the presence and absence of *F. oxysporum* VCs. This experiment was performed as outlined in Figure 1B. After inoculating a plug of *Trichoderma* culture on sterilized cellophane membrane (Paper Mart, Orange, CA, United States) overlaid on PDA, each plate was sandwiched with a plate of 5-day-old *F. oxysporum* culture or a un-inoculated PDA plate (Control). After 33 h (for *T. harzianum* and *T. asperellum*) or 40 h (for *T. virens* and *T. viride*) of co-cultivation, cellophane membrane along with *Trichoderma* culture was removed. The harvested culture was immediately frozen in liquid nitrogen and stored at −80°C until RNA extraction. The antifungal activity of molecules secreted into the medium by *Trichoderma* after each treatment was measured by inoculating a plug of *F. oxysporum* culture on the medium. Colony diameter of *F. oxysporum* was measured after 3 days for *T. harzianum* and *T. asperellum* and 5 days for *T. virens* and *T. viride* due to stronger inhibition by the latter two. Relative colony diameter after each VC treatment (%) was calculated using (D_treatment_/D_control_) × 100, where D_control_ and D_treatment_ indicated the colony diameters of *F. oxysporum* in the control and VC treatments, respectively.

Whether *F. oxysporum* VCs affect the amount/activity of antifungal VCs released by *Trichoderma* was determined as follows. After co-cultivation as described above, each *Trichoderma* plate was sandwiched with a new plate inoculated with a plug of *F. oxysporum* culture. The colony diameter of *F. oxysporum* was measured 3 days later. Each treatment consisted of three biological replicates and was repeated three times.

### VC-mediated recognition of *Trichoderma* by *F. oxysporum*

The ability of *F. oxysporum* to sense *Trichoderma* VCs was evaluated similarly (Figure 1B). Each plate of 3-day-old *F. oxysporum* culture on sterilized cellophane membrane was sandwiched with a 1-day-old *Trichoderma* culture or control (un-inoculated PDA) plate. After 40 h of co-cultivation, *F. oxysporum* culture was removed, and a plug of *T. virens* culture was inoculated. Colony diameter was measured 2 days later. Each treatment was performed in three biological replicates and repeated three times.

### Determination of the protein permeability of cellophane membrane

First, we placed four drops of 5 μL bovine serum albumin (Amresco, Solon, OH) solution at the concentrations of 0.01, 0.1, and 1 mg/ml on cellophane membrane overlaid on PDA and incubated the plate overnight. Drops of the same solutions were applied to PDA without cellophane membrane. These plates were stained using 20 mL Coomassie blue solution (Bio-Rad Laboratories, Richmond, CA, United States) for 1 h. Second, PDA plate containing a plug of *Trichoderma* culture inoculated on cellophane membrane was incubated for 40 h. After removing the cellophane membrane, the plate was stained to detect the presence of secreted proteins. We also compared the activity of antifungal molecules permeated through the cellophane and dialysis (Spectra/Por® 3 with the molecular weight cut off of 3.5 kD; Spectrum Laboratories, Rancho Dominguez, CA, United States) membranes. A plug of *Trichoderma* culture was inoculated on each membrane on PDA. After 40 h of incubation, the membrane with *Trichoderma* culture was removed, and a plug of *F. oxysporum* culture was inoculated on the same medium to measure the degree of growth inhibition. This comparison was repeated twice with three biological replicates.

### Analysis of VCs produced by *F. oxysporum* and *Trichoderma*

A culture dish of 10 cm in diameter and 4 cm in height (SPL Life Sciences, South Korea) that contains 250 mL PDA was used to extract *F. oxysporum* VCs (Supplementary Figure 1). A hole (5 mm in diameter) on its lid, created using a flamed cork borer, was sealed using a piece of tape. A PDA block beneath the hole was removed before inoculating *F. oxysporum*. We cultured *F. oxysporum* for 14 days before extracting headspace VCs. *Trichoderma* was cultured on PDA slant in a 15 ml glass vial for 3 days. Extraction of headspace VCs (two biological replicates) was performed as previously described (Li et al., 2018). A solid phase micro-extraction (SPME) fiber with 50/30 μm divinylbenzene/carboxen/polydimethylsiloxane coating was conditioned at 230°C for 1 h before VC extraction to remove any VCs from the fiber. Headspace VCs released from PDA without fungal culture were analyzed to identify VCs derived from the medium. VCs in the headspace were adsorbed for 1 h. The SPME fiber was desorbed at 230°C for 10 min in the split/splitless injector port (set at the splitless mode) of QP 2010 Ultra GC-MS system (Shimadzu, Japan) equipped with an Rtx-Wax capillary column (60 m × 0.25 mm × 0.25 μm, RESTEK, Bellefonte, PA). The temperature program was 35°C for 5 min, ramped to 230°C at 5°C/min (*F. oxysporum*) or 2°C/min (*Trichoderma* spp., for better separation of compounds), and kept for 15 min at 230°C. We applied helium (carrier gas) at the constant flow rate of 1 mL/min. The injector, transfer line, and ion source temperatures were set at 230°C. The electron ionization source was set at 70 eV. Data acquisition started at 13 min, and the mass scan range was 35–500 m/z. An alkane standard mixture (1 μL; C_7_-C_40_ containing 100 mg/L of each alkane in n-hexane) was analyzed to calculate the retention indices of individual VCs detected. We tentatively identified individual VCs by comparing their mass spectral profiles and retention indices with those archived in the NIST 11 library using the version 2.7 of the AMDIS (automated mass spectral deconvolution and identification system) software. We used the following criteria for compound identification: a match factor of ≥90% between sample spectra and reference spectra and ≤ ± 2% deviation of experimentally determined retention index values from those in the literature. The identity of the following compounds, which were produced by all *F. oxysporum* strains analyzed, was confirmed using commercially available standards: 1-hexanol, 3-methyl-1-butanol, isopentyl acetate, and 2-phenylethyl alcohol (Sigma-Aldrich) and 4-ethylanisole (Alfa Aesar, Haverhill, MA, United States). We used 20 μL of each compound diluted using hexane for GC-MS analysis.

### RNA extraction and quantitative real-time PCR (qPCR) analysis

To determine whether *F. oxysporum* VCs affect the expression of biocontrol-associated genes in *Trichoderma*, we extracted total RNAs from *Trichoderma* cultures harvested after treating with *F. oxysporum* VCs. The experiment was performed once with three biological replicates. Each biological replicate consisted of two *Trichoderma* cultures independently treated with *F. oxysporum* VCs or PDA. After treating extracted RNAs with DNase I (Ambion, Foster City, CA), they were purified using Qiagen RNeasy kit (Qiagen, Valencia, CA). Concentration and quality of purified RNAs were assessed using the A260/A280 and A260/230 absorbance ratios and gel electrophoresis. RNAs (1 μg) were reverse transcribed to cDNAs in a 20 μL reaction (Applied Biosystems, Foster City, CA, United States). Sequences of the 13 genes used for gene expression analysis (Table 2) were mined from genome sequences of *T. virens* Gv29-8 (V2.0) and *T. harzianum* CBS 226.95 (V1.0) available at http://genome.jgi.doe.gov/. The primers used (Supplementary Table 1) were designed using Primer 3 (http://primer3.ut.ee/). Primer specificity was tested using genomic DNA as a template. Each PCR reaction with three technical replicates was performed using Power SYBR® Green kit (Applied Biosystems) and consisted of 12.5 μL PCR master mix, 1 μL each of forward and reverse primers (10 μM), 2 μL diluted cDNAs (1:5 dilution), and 8.5 μL UltraPure™ DNase/RNase-free distilled water. qPCR conditions were: (a) one cycle of 10 min at 95°C, (b) 40 cycles of 30 s at 95°C, 30 s at 60°C, and 45 s at 72°C, (c) one cycle of 1 min at 95°C and 30 s at 55°C, and (d) final ramp to 95°C to check primer dimerization and nonspecific amplification. We used sterile water as no DNA control and RNAs after DNase treatment to confirm the absence of genomic DNA. Genes encoding elongation factor 1-α and β-actin were used to normalize gene expression (Seidl et al., 2009; Liu et al., 2010). Relative expression levels of individual genes were calculated from the threshold cycle using the Pfaffl method for efficiency correction (Pfaffl, 2001). Amplification efficiency was calculated as previously described (Rasmussen, 2001) using serially diluted cDNA samples (1, 1/5, 1/25, 1/125, and 1/625), which showed values between 90% and 110%.

**Table 2:** *Trichoderma* genes chosen for expression analysis.

**Gene (abbreviation)^a^**	**Function**	**Mode of induction^b^**	**Homologous gene in ref. genome^c^**	
			***T. virens***	***T. harzianum***
chitinase 33 (*chit*)	Cell wall degradation	AC	189743	529621
endochitinase 42 (*endo*)	Cell wall degradation	BC	218832	101028
β-1,3-endoglucanase (*bgn*)	Cell wall degradation	AC	231316	485240
trypsin-like protease pra1 (*pra*)	Protein degradation	AC	181449	526221
subtilisin-like protease prb1 (*prb*)	Protein degradation	AC	186844	110777
QID74 protein (*qid*)	Cell wall integrity	BC	230780	509414
small heat-shock protein Hsp26 (*hsp*)	Posttranslational processing	AC	195080	8037
acyl-CoA synthetase (*acs*)	Lipid metabolism	BC	203203	479794
anthranilate synthase component II (*ans*)	Amino acid metabolism	BC	193889	534341
polyketide synthase (*pks)*	Secondary metabolism	AC	204589	521432
terpene synthase (*tps*)	Terpene synthesis	unknown	141474	523651
translation elongation factor 1-α (*tef*)	Housekeeping gene	–	216092	12328
β-actin (*act*)	Housekeeping gene	–	229062	510028

a *With the exception of tps, tef, and act, they were chosen because their expression was reported to be induced during mycoparasitism*.

b *AC, gene expression induced after physical contact. BC, gene expression induced both before and after physical contact. –, no induction*.

c *Transcript IDs of the genes in T. virens Gv29-8 v2.0 and T. harzianum CBS 226.95 v1.0 are shown*.

### Statistical analysis

One-way analysis of variation (ANOVA) was performed using Minitab 18 (Minitab Inc., State College, PA, United States). The significance of each treatment was determined using the *F-*value. When a significant *F-*test was observed, separation of the means was carried out using Fisher's test. Statistical significance was determined at *P* = 0.05.

## Results

### Sandwiched culture plates were used to study how *Trichoderma* BCAs and *F. oxysporum* interact via VCs

To prevent physical contact and exposure to secreted molecules diffused through medium while studying how VCs from each side affect the other, we initially evaluated I plate. This plate contains two compartments separated by a central divider and has been used for studying how fungal VCs affect plants (Bitas et al., 2015; Li and Kang, 2018; Li et al., 2018). However, because *Trichoderma* jumped over the divider even after we removed a strip of medium along the divider in both compartments, we used a method reported by Dennis and Webster (1971). We did not detect any incidence of *F. oxysporum* spores dropping to the bottom plate during co-cultivation, which was checked multiple times by placing a PDA plate at the bottom.

We investigated the following questions via the experiments outlined in Figure 1: (a) do VCs released by *Trichoderma* inhibit *F. oxysporum* growth and vice versa?; (b) does *Trichoderma* recognize the presence of *F. oxysporum* by sensing volatile cues from *F. oxysporum* and vice versa?; and (c) does VC-mediated recognition cause molecular changes potentially associated with biocontrol? All *Trichoderma* strains analyzed were isolated from commercial biocontrol products to facilitate future studies on the role of VC-mediated interactions in biocontrol. Thirteen *F. oxysporum* strains (Table 1), corresponding to seven *formae speciales*, were used.

### Both *Trichoderma* and *F. oxysporum* produced antifungal VCs

**Figure 2:**
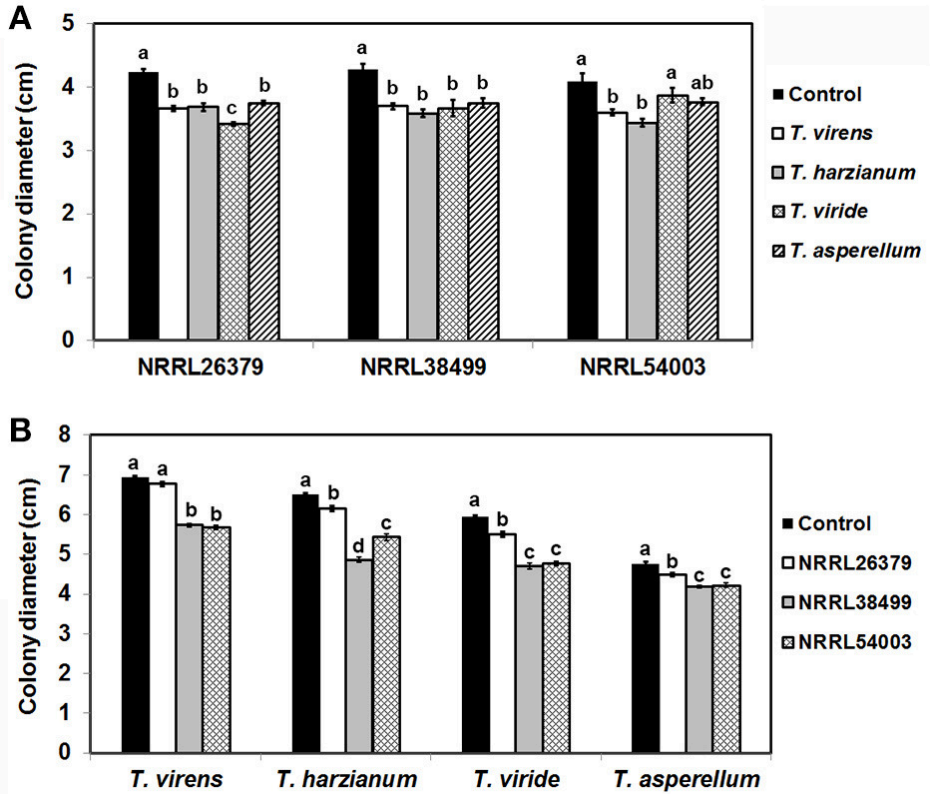
Inhibitory effect of volatile compounds (VCs) produced by *Trichoderma* and *Fusarium oxysporum*. After sandwiching culture plates of *Trichoderma* and *F. oxysporum* as described in Figure 1A, colony diameters of **(A)** three *F. oxysporum* strains after co-cultivation with four *Trichoderma* spp. and **(B)** four *Trichoderma* spp. after co-cultivation with three *F. oxysporum* strains were measured. Control treatments for **(A,B)** consisted of *F. oxysporum* and *Trichoderma* culture plates, respectively sandwiched with un-inoculated PDA plates. Values shown correspond to the mean ± standard deviation (SD) of data from three replicates. Different letters indicate significant differences based on Fisher's test at *P* = 0.05.

**Figure 3:**
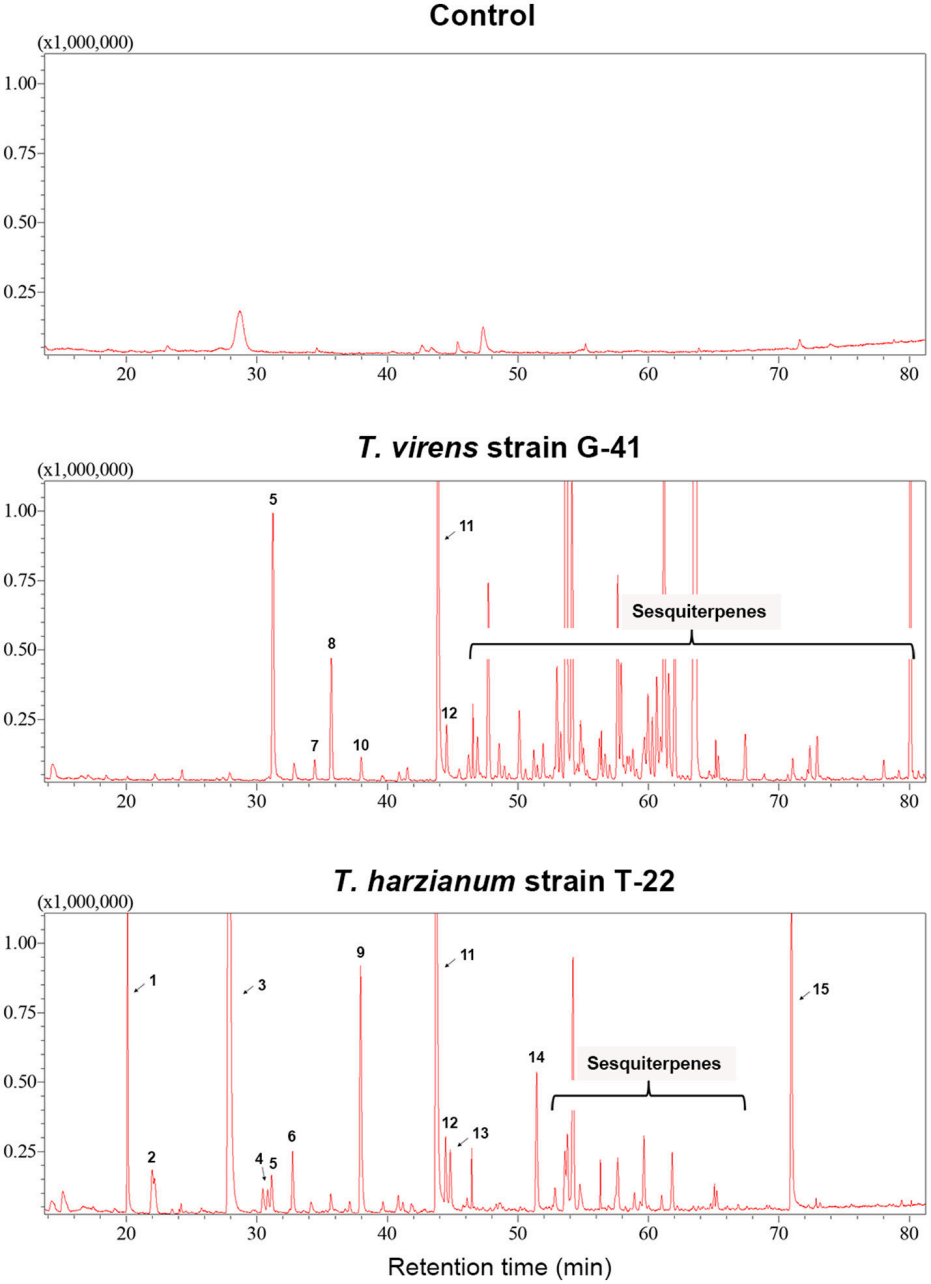
VCs produced by *T. virens* and *T. harzianum*. Headspace volatiles extracted from Control (PDA only) and cultures of *T. virens* strain G-41 and *T. harzianum* strain T-22 on PDA were separated using GC. The identity of compound corresponding to each numbered peak is shown in Table 3. X-axis, retention time (in minutes); Y-axis, intensity (counts per second).

*Trichoderma* BCAs secrete metabolites inhibitory to diverse pathogens (Reino et al., 2007; Vinale et al., 2014; Zeilinger et al., 2016), and some such metabolites are volatile (Amin et al., 2010; Meena et al., 2017). Here, we tested if VCs produced by four *Trichoderma* spp. inhibit *F. oxysporum* and vice versa (Figure 2). VCs produced by all *Trichoderma* spp. significantly inhibited the growth of three *F. oxysporum* strains (Figure 2A)*. T. viride* VCs inhibited NRRL26379 (f. sp. *radicis-lycopersici*) most, whereas *T. harzianum* VCs inhibited NRRL38499 (f. sp. *lycopersici*) and NRRL54003 (f. sp. *lycopersici*) more than NRRL26379. To identify candidate VCs inhibitory to *F. oxysporum*, we analyzed VCs produced by 3-day-old *T. harzianum* and *T. viride* cultures. Most of the identified compounds, corresponding to alcohols, acids, esters, ketones, and sesquiterpenes, are species-specific (Figure 3 and Table 3). While *T. harzianum* emitted several alcohols, including 2-methyl-1-propanol, 3-methyl-1-butanol, 1-pentanol, 1-hexanol, 1-heptanol, 1-octanol, and 2-phenylethyl alcohol, *T. virens* produced only 3-methylcyclopentanol. Only compounds that were produced by both species are 1-octen-3-ol, 3-octanone, and acetic acid. It was evident that *T. virens* produces more sesquiterpenes in both the number and amount than *T. harzianum*.

**Table 3:** VCs produced by *T. virens* and *T. harzianum*.

**#^a^**	**Putative identity^b^**	**Formula**	**RIcal^c^**	**RIcal^d^**	***Trichoderma*** **analyzed**^**e**^	
					***T. virens***	***T. harzianum***
1	2-Methyl-1-propanol	C_4_H_10_O	1,071	1,068		√
2	Isopentyl acetate	C_7_H_14_O_2_	1,103	1,102		√
3	3-Methyl-1-butanol	C_5_H_12_O	1,189	1,190		√
4	1-Pentanol	C_5_H_12_O	1,132	1,132		√
5	3-Octanone	C_8_H_16_O	1,236	1,236	√	√
6	Acetoin	C_4_H_8_O_2_	1,259	1,259		√
7	1-Octen-3-one	C_8_H_14_O	1,282	1,282	√
8	Methyl (2E)-4,4dimethyl-2-pentenoate	C_8_H_14_O_2_	1,285	–	√
9	1-Hexanol	C_6_H_14_O	1,333	1,335		√
10	3-Methylcyclopentanol	C_6_H_12_O	1,333	1,342	√
11	Acetic acid	C_2_H_4_O_2_	1,418	1,418	√	√
12	1-Octen-3-ol	C_8_H_16_O	1,428	1,428	√	√
13	1-Heptanol	C_7_H_16_O	1,434	1,436		√
14	1-Octanol	C_8_H_18_O	1,537	1,537		√
15	2-Phenylethyl alcohol	C_8_H_10_O	1,876	1,876		√

a *The numbers correspond to the GC peaks marked in Figure 3. We numbered them according to the order of elution*.

b *Putative identity of VCs present in the headspace of 3-day-old Trichoderma cultures is shown. Only the compounds that were present in two biological replicates are listed. Mass spectra and retention index of each compound were compared with reference data in the NIST library for identification*.

c *Linear Retention Index (RI) of each compound determined using a mixture of alkane standards (C_7_-C_40_)*.

d *Linear RI of each compound found in the literature. – (Linear RI of these compounds are not available in the literature)*.

e *√ denotes the presence of each compound*.

We tested whether *F. oxysporum* also releases VCs inhibitory to *Trichoderma* (Figure 2B). Because *F. oxysporum* grows much more slowly than *Trichoderma*, we initiated *F. oxysporum* culture 5 days before co-cultivation to ensure that *F. oxysporum* strains produce sufficient amounts of VCs. VCs from all three *F. oxysporum* strains inhibited *T. harzianum, T. viride*, and *T. asperellum* with VCs from NRRL38499 and NRRL54003 causing stronger inhibition than VCs from NRRL26379. VCs from NRRL38499, and NRRL54003 but not NRRL26379 significantly inhibited *T. virens*.

### *F. oxysporum* VCs increased the amount/activity of antifungal molecules secreted by *T. virens*

**Figure 4:**
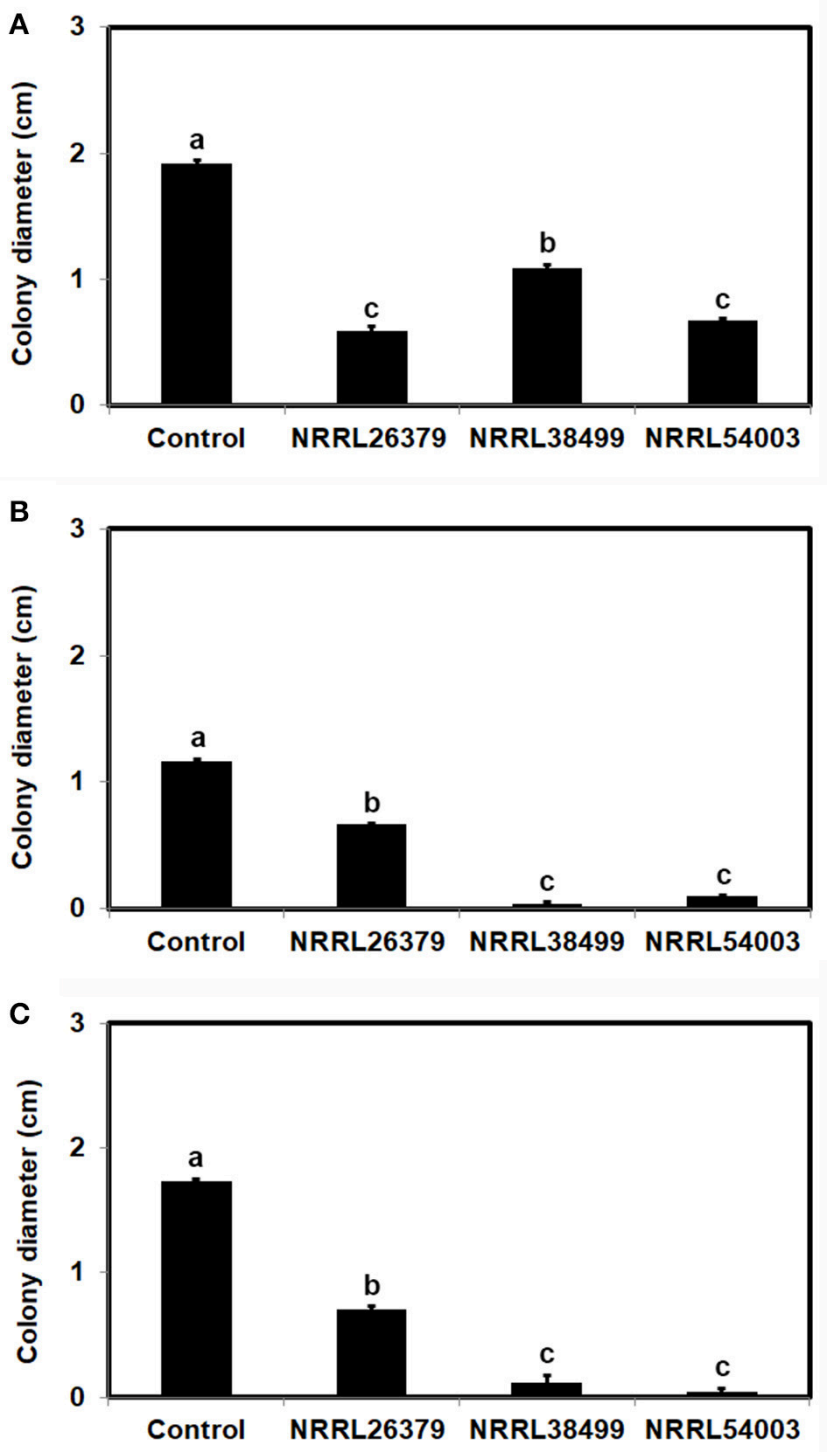
Increased amount/activity of antifungal molecules secreted by *T. virens* in response to *F. oxysporum* VCs. *T. virens* strain G-41 inoculated on cellophane membrane overlaid on PDA was co-cultivated with three *F. oxysporum* strains as well as un-inoculated PDA (Control) for 40 h. After removing the cellophane membrane with *T. virens* culture, a plug of **(A)** NRRL54003, **(B)** NRRL26379, or **(C)** NRRL38499 culture was inoculated. Colony diameters after 5 days of incubation are shown. Values shown correspond to the mean ± SD of data from three replicates. Different letters indicate significant differences based on Fisher's test at *P* = 0.05.

To test the hypothesis that *Trichoderma* recognizes *F. oxysporum* by sensing VCs from *F. oxysporum* and prepares for attack, we determined if *F. oxysporum* VCs induce the amount/activity of antifungal molecules secreted by *T. virens*. First, *T. virens* was cultured on sterilized cellophane membrane overlaid on PDA to prevent its hyphae from reaching medium but allow the passage of secreted molecules to the medium during co-cultivation with *F. oxysporum*. After removing the membrane along with *T. virens* culture, *F. oxysporum* strain NRRL54003 was inoculated on the same medium to measure its growth. Exposure to VCs from NRRL26379, NRRL38499, and NRRL54003 caused significantly higher growth inhibition than control treatments, but *T. virens* responded more strongly to VCs from NRRL26379 and NRRL54003 than VCs from NRRL38499 (Figure 4A). The growth of two other *F. oxysporum* strains was also measured to determine whether the increased antifungal activity caused by VCs from individual strains similarly affects them (Figures 4B,C). Their growth was also significantly inhibited, but the degree of inhibition looked different from that observed with NRRL54003 as a tester. The degree of inhibition after exposure to NRRL26379 VCs was significantly lower than that caused by VCs from NRRL38499 and NRRL54003 (Figure 4). We also evaluated whether *F. oxysporum* VCs increase the production of antifungal VCs by *T. virens*. Results from the VC and control treatments were indistinguishable(Supplementary Figure 2).

### *F. oxysporum* VCs induced the amount of antifungal metabolites secreted by *Trichoderma*

To determine whether the increased antifungal activity of *T. virens* secretome caused by *F. oxysporum* VCs was due to secreted metabolites, proteins or a combination of both, we evaluated the permeability of proteins through the cellophane membrane used in our work (Supplementary Figure 3). Although the two experiments shown in Supplementary Figure 3A could not directly answer this question, they suggested that proteins could not readily pass through the membrane. Besides, when we cultured *T. virens* on dialysis membrane (with the molecular weight cut off of 3.5 kD), the degree of inhibition was not significantly different from that based on cellophane membrane (Supplementary Figure 3B), suggesting that the increased antifungal activity after the exposure to *F. oxysporum* VCs (Figure 4) was mainly due to metabolites secreted into the medium.

### *T. virens* and *T. viride* but not *T. asperellum* responded to VCs from diverse *F. oxysporum* strains

We employed 10 additional strains corresponding to five *formae speciales* (Table 1) to determine *T. virens*' response. VCs produced by all of them significantly induced the amount of antifungal metabolites secreted by *T. virens* (Table 4). VCs produced by NRRL38487 (f.sp. *conglutinans*), NRRL37616 (f.sp. *pisi*) and NRRL26029 (f.sp. *cubense*) caused the highest degree of induction, completely blocking *F. oxysporum* growth. Similarly, VCs from all 13 strains significantly induced the amount of antifungal metabolites secreted by *T. viride* with VCs from five strains (9605, W6-1, NRRL38272, NRRL37611, and NRRL36118) causing complete growth inhibition (Table 4). However, none of them induced the amount of antifungal metabolites secreted by *T. asperellum* (Table 4).

**Table 4:** Effect of VCs produced by diverse *F. oxysporum* strains on the secretion of antifungal metabolites by four *Trichoderma* spp.

***F. oxysporum* strains**	**Relative colony diameter (%)**^**a**^			
	***T. virens***	***T. viride***	***T. harzianum***	***T. asperellum***
NRRL26379	30.7 ± 2.6 (d)	55.8 ± 2.4 (b)	–	–
NRRL38499	56.9 ± 1.9 (a)	25.7 ± 3.1 (e)	–	–
NRRL54003	35.0 ± 2.2 (c)	24.8 ± 0.6 (e)	–	–
9605	15.2 ± 3.8 (g)	no growth (g)	–	–
W61	19.6 ± 1.3 (f)	no growth (g)	–	–
NRRL38272	24.1 ± 2.6 (e)	no growth (g)	–	–
NRRL38487	no growth (h)	27.0 ± 1.7 (d)	59.6 ± 3.1 (a)	–
NRRL37611	25.0 ± 2.1 (e)	no growth (g)	–	–
NRRL37616	no growth (h)	27.0 ± 2.1 (d)	61.4 ± 2.0 (a)	–
NRRL26029	no growth (h)	3.0 ± 1.8 (f)	59.9 ± 1.5 (a)	–
NRRL36118	17.9 ± 3.1 (g)	no growth (g)	–	–
NRRL22518	34.0 ± 1.0 (c)	40.0 ± 2.5 (c)	–	–
NRRL22519	37.5 ± 2.1 (b)	68.0 ± 2.9 (a)	–	–

a *After exposure of individual Trichoderma strains to VCs from the listed F. oxysporum strains and Control (Trichoderma co-cultivated with un-inoculated PDA plate), F. oxysporum strain NRRL54003 was used to assess the degree of growth inhibition caused by secreted Trichoderma metabolites. Relative colony diameter (%) after each treatment was calculated using (D_treatment_/D_control_) × 100, where D_control_ and D_treatment_ indicated the colony diameters of F. oxysporum in control and VC treatments, respectively. Values shown correspond to the mean ± standard deviation (SD) of data from three replicates. Different letters indicate significant differences between treatments, according to Fisher's test at P = 0.05. – indicates no induction*.

### *T. harzianum* responded to VCs from only some *F. oxysporum* strains

**Figure 5:**
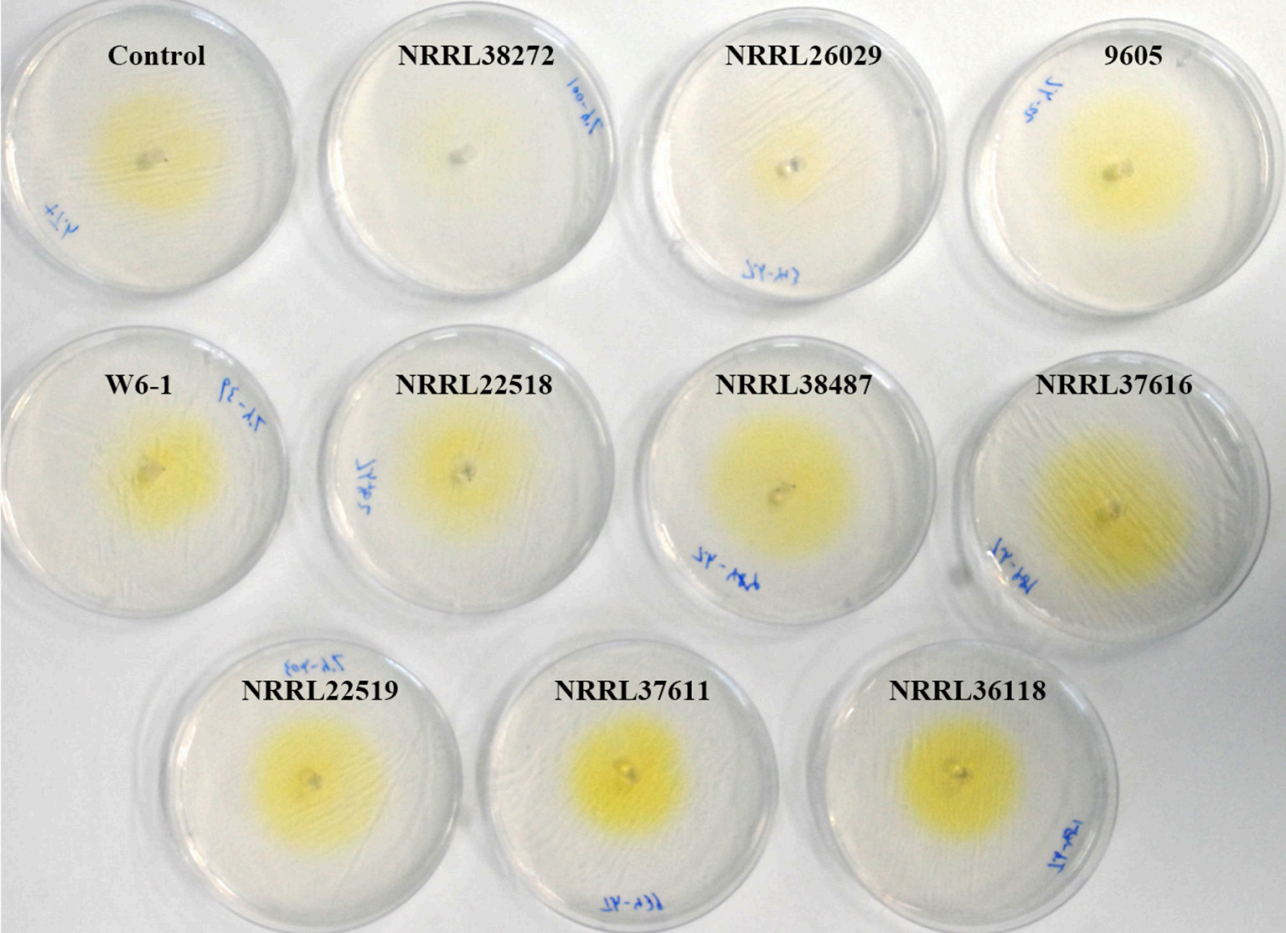
Effect of *F. oxysporum* VCs on the secretion of yellow pigment by *T. harzianum. T. harzianum* strain T-22 cultured on cellophane membrane overlaid on PDA was co-cultivated with 10 *F. oxysporum* strains and un-inoculated PDA (Control) for 33 h. After removing the cellophane membrane holding T-22 culture, plates were photographed (arranged based on the amount of pigment released).

**Figure 6:**
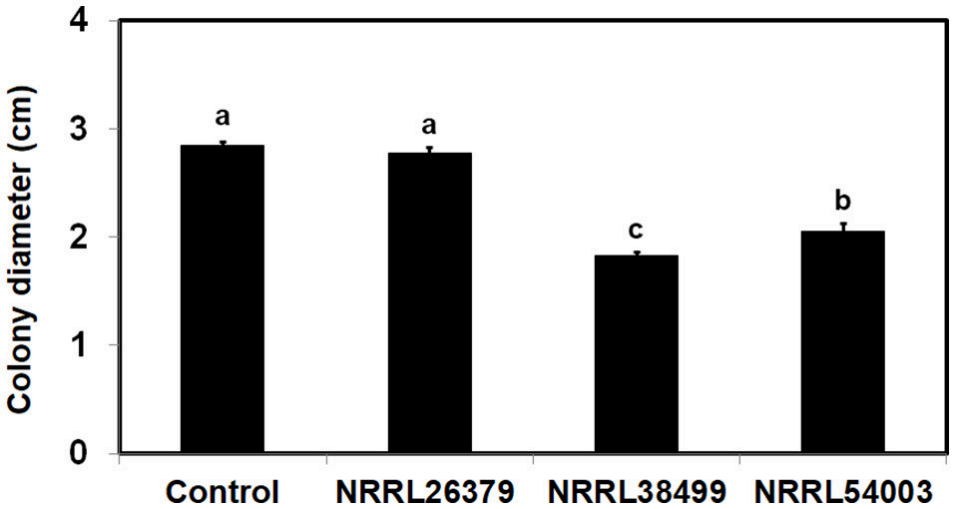
Effect of *F. oxysporum* VCs on the amount/activity of antifungal metabolites secreted by *T. harzianum*. *T. harzianum* strain isolated from Custom GP was exposed to VCs from three *F. oxysporum* strains and un-inoculated PDA (Control) for 33 h. After removing the cellophane membrane along with *T. harzianum* culture, one plug of NRRL54003 culture was inoculated on each plate. Colony diameters after 3 days of incubation are shown. Values shown correspond to the mean ± SD of data from three replicates. Different letters indicate significant differences based on Fisher's test at *P* = 0.05.

VCs from only NRRL38487, NRRL37616, and NRRL26029, which caused the highest induction in *T. virens*, significantly induced the amount of antifungal metabolites secreted by *T. harzianum* strain T-22 (Table 4). The degree of induction in *T. harzianum* was significantly lower than those observed in *T. virens* and *T. viride*. During this experiment, we observed that the amount of yellow pigment secreted by *T. harzianum* was also affected by *F. oxysporum* VCs (Figure 5). VCs from NRRL38272 and NRRL26029 appeared to suppress its production, while VCs from NRRL37616, NRRL22519, NRRL37611, and NRRL36118 increased its production. However, the amount secreted did not correlate with the degree of *F. oxysporum* inhibition. We determined if a different *T. harzianum* strain responds to VCs from some of the *F. oxysporum* strains that did not induce the secretion of antifungal metabolites by *T. harzianum* strain T-22. This *T. harzianum* strain responded to VCs from NRRL38499 and NRRL54003 but not NRRL26379 (Figure 6).

### Expression of some biocontrol-associated genes in *Trichoderma* was affected by *F. oxysporum* VCs

**Figure 7:**
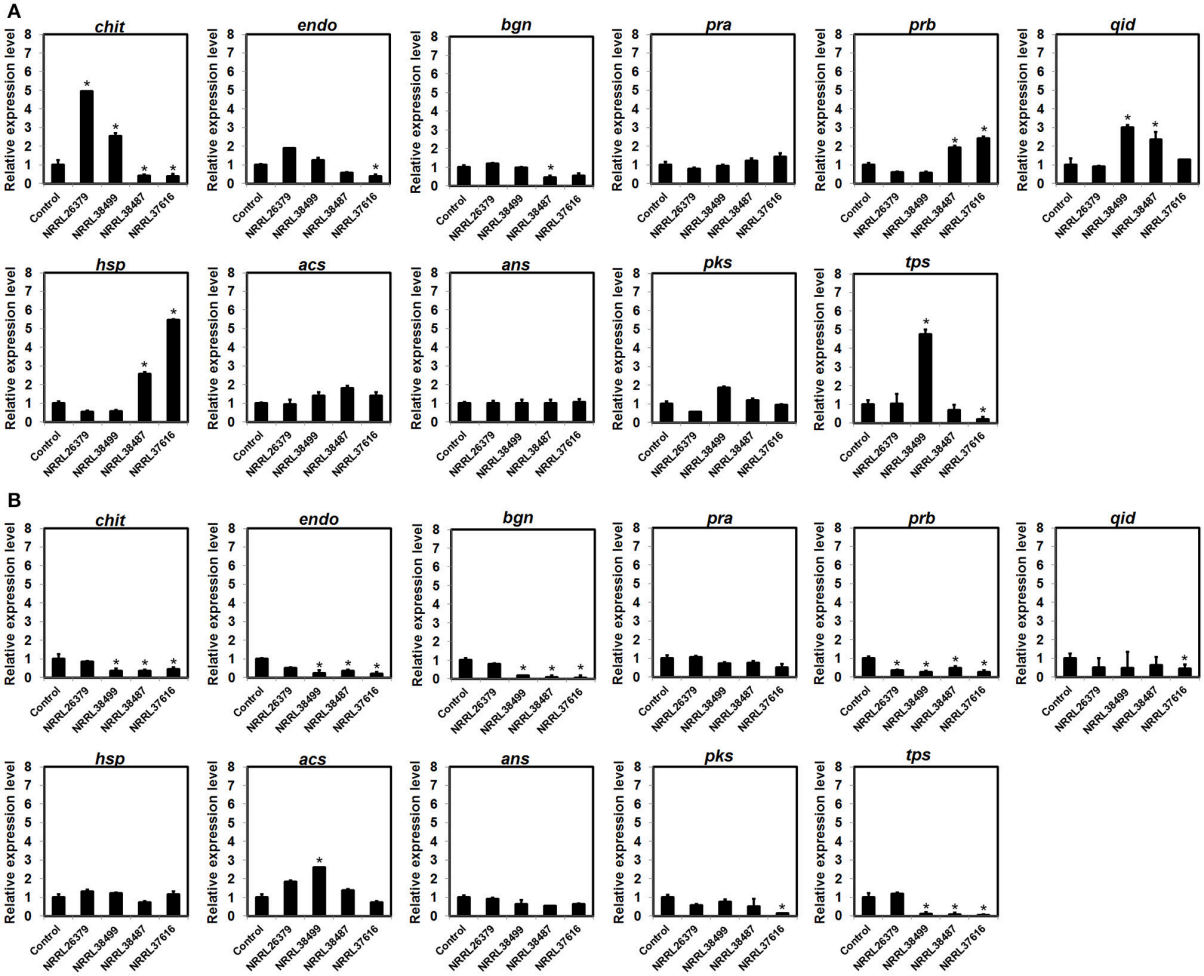
Expression patterns of biocontrol-associated genes after exposure to *F. oxysporum* VCs. Transcripts from 11 genes in **(A)** *T. virens* strain G-41 and **(B)** *T. harzianum* strain T-22 were quantified after co-cultivation with four *F. oxysporum* strains (NRRL26379, NRRL38499, NRRL38487, and NRRL37616) for 40 h and 33 h, respectively. Transcripts from the elongation factor 1-α and β-actin genes were used as internal references to normalize the level of transcripts for individual genes. Control corresponded to *Trichoderma* co-cultivated with un-inoculated PDA. Values shown correspond to the relative expression ± SD of data from three replicates. Gene identity based on the abbreviation scheme shown in Table 2 is indicated. * indicates up-regulation (relative expression level >2) or down-regulation (relative expression level < 0.5).

To investigate if *F. oxysporum* VCs affect the expression of biocontrol-associated genes, we analyzed transcripts from 11 genes in *T. virens* and *T. harzianum* after co-cultivation with NRRL26379, NRRL38499, NRRL38487, and NRRL37616. VCs from NRRL38487 and NRRL37616 induced the secretion of antifungal metabolites much more strongly than VCs from NRRL38499 and NRRL26379 in both *T. virens* and *T. harzianum* (Table 4). Ten genes (Table 2) were chosen because their expression increased upon pathogen confrontation (Samolski et al., 2009; Seidl et al., 2009; Vieira et al., 2013; Yao et al., 2016). Four genes, including *endo* (endochitinase 42), *qid* (QID74)*, acs* (acyl-CoA synthetase), and *ans* (anthranilate synthase component II), showed increased transcription even before physical contact (Seidl et al., 2009; Vieira et al., 2013), suggesting that airborne signals or small molecules diffused through medium might induce their expression. The terpene synthase (*tps*) gene was hypothesized to be involved in biocontrol (Kubicek et al., 2011). VCs from some strains up-regulated five *T. virens* genes (Figure 7A). Expression of *chit* (chitinase 33) was significantly higher after exposure to VCs from NRRL26379 and NRRL38499, but it was down-regulated by VCs from NRRL38487 and NRRL37616. Whereas VCs from NRRL38487 and NRRL37616 induced the expression of *prb* (subtilisin-like protease) and *hsp* (heat-shock protein), VCs from NRRL26379 and NRRL38499 slightly suppressed their expression. VCs from NRRL38499 and NRRL38487 significantly increased the level of *qid* transcripts. VCs from NRRL38499 significantly induced the expression of *tps*, where VCs from NRRL37616 suppressed its expression. VCs from NRRL37616 and NRRL38487 suppressed the expression of *endo*) and *bgn* (β-1, 3-endoglucanase), respectively. Expression of four genes (*pra, acs, ans*, and *pks*) did not appear significantly affected by any treatments. In *T. harzianum* (Figure 7B), only *acs* was induced by VCs from NRRL38499. Expression of *prb* was down-regulated by VCs from all four strains. VCs from NRRL38499, NRRL38487, and NRRL37616 appeared to suppress the expression of *chit, endo, bgn*, and *tps*. VCs from NRRL37616 suppressed the expression of *qid* and *pks*.

### Seven *F. oxysporum* strains commonly produced six VCs

Increased secretion of antifungal metabolites by *T. virens* and *T. viride* in response to VCs from diverse *F. oxysporum* strains (Table 4) suggested that commonly produced compound(s) might be responsible. To identify such compounds, we analyzed VCs produced by seven strains corresponding to five *formae speciales* (Table 5). Initially, they were cultured in 15 mL glass vials to extract VCs. However, many compounds were unidentifiable because the amount of VCs captured was too small. We increased the concentration of headspace VCs by expanding the culture area while reducing the headspace volume (Supplementary Figure 1). Collectively, 28 VCs were identified (Table 5). Six compounds, including 3-methyl-1-butanol, 1-hexanol, 2-phenylethyl alcohol, isopentyl acetate, 4-ethylanisole, and α-cedrene, were produced by all strains. Two compounds, including β-caryophyllene and D-germacrene, were emitted by only one strain (NRRL38487 and NRRL54003, respectively). Two or more strains produced the remaining compounds.

**Table 5:** VCs produced by seven *F. oxysporum* strains.

**Putative identity^a^**	**RIcal^b^**	**RIref^c^**	***F. oxysporum*** **strains analyzed (NRRL#)**^**d**^						
			**26379**	**38499**	**54003**	**38272**	**38487**	**37616**	**26029**
**ALCOHOLS**									
2-Methyl-1-propanol	1,073.1	1,065		√	√	√	√	√	√
3-Methyl-1-butanol	1,182.9	1,180	√	√	√	√	√	√	√
1-Hexanol	1,324.1	1,324	√	√	√	√	√	√	√
2-Phenylethyl alcohol	1,867.7	1,868	√	√	√	√	√	√	√
**KETONES**									
2-Heptanone	1,159.4	1,160	√					√
5-Ethyl-4-methyl-3-heptanone	1,301.2	–		√			√	
2-Nonanone	1,374.9	1,375				√		√
3-Ethyl-2-undecanone	1,530.4	–	√	√		√		√
(E)-6-Methyl-3,5-heptadienone	1562.2	1,561		√		√		√	√
**ESTERS**									
Isopentyl acetate	1,107.6	1,108	√	√	√	√	√	√	√
Ethyl 3-methyl-2-butenoate	1,209.6	1,216.4		√		√	√	
Hexyl acetate	1,261.7	1,258	√		√	√		√	√
Heptyl acetate	1,359.7	1,361	√	√				√	√
Octyl acetate	1,463.8	1,464	√		√	√	√	√	√
Phenethyl acetate	1,782.9	1,783	√	√	√		√	√	√
**ETHERS**									
4-Ethylanisole	1,530.9	1,550	√	√	√	√	√	√	√
1-Ethenyl-4-methoxybenzene	1,647.1	1,670	√	√	√		√	√	√
**SESQUITERPENES**									
α-Cedrene	1,583.3	1,582	√	√	√	√	√	√	√
β-Caryophyllene	1,607.7	1,607					√	
β-Cedrene	1,610	1,611	√	√	√	√			√
(E)-β-Famesene	1,663.3	1,684.2	√	√	√	√	√		√
Acoradien	1,681	1689.1	√	√	√	√	√		√
D-Germacrene	1,716.8	1,716			√			
α-Zingiberene	1,722.4	1,715	√	√		√	√		√
β-Bisabolene	1,731.3	1,725	√	√		√	√		√
β-Curcumene	1,743.6	–	√	√		√	√		√
β-Sesquiphellandrene	1,768.9	1,768	√	√		√			√
α-Acorenol	2,125.5	–	√	√	√	√	√		√

a *Putative identity of VCs present in the headspace of 14-day-old F. oxysporum culture on PDA is shown. Only the compounds that were detected in two biological replicates are listed. Mass spectra and retention index (RI) of each compound were compared with reference data in the NIST library for identification*.

b *Linear RI of each compound was determined using a mixture of alkane standards (C_7_-C_40_)*.

c *Linear RI of each compound found in the literature is shown. – (Linear RI of these compounds is not available in the literature)*.

d *√ denotes the presence of each compound*.

### *F. oxysporum* also responded to *trichoderma* VCs by increasing the secretion of its antifungal metabolites

**Figure 8:**
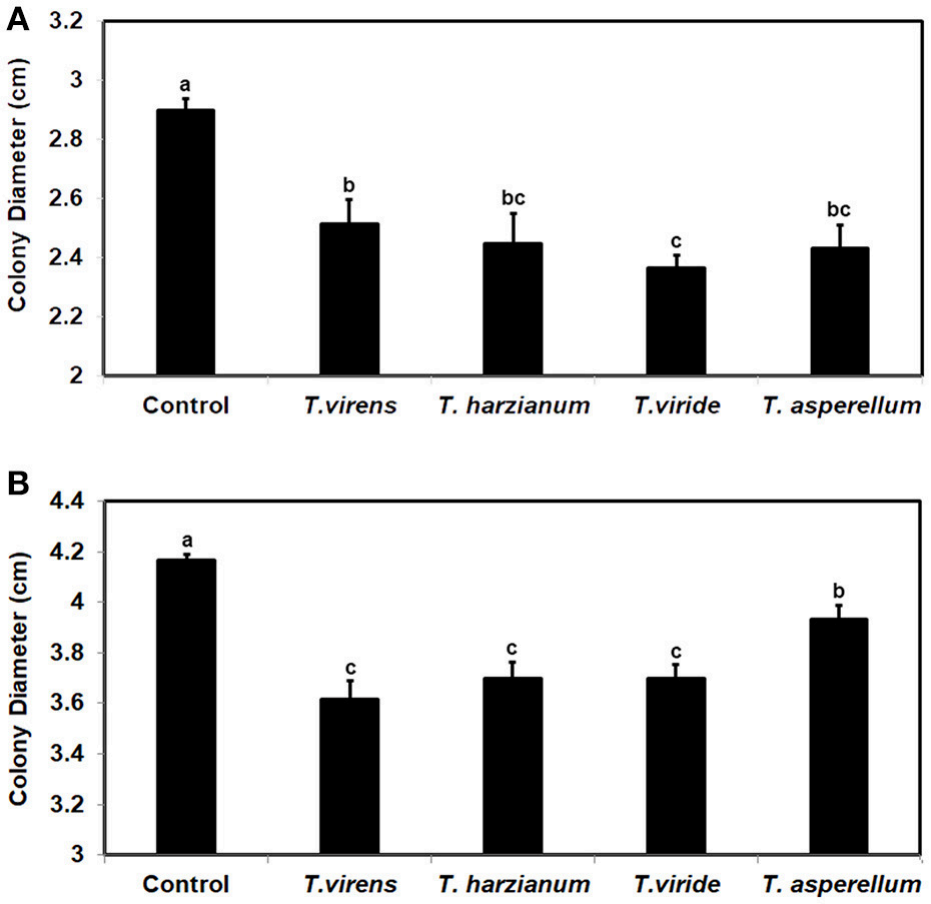
Increased secretion of antifungal metabolites by *F. oxysporum* in response to *Trichoderma* VCs. *F. oxysporum* strains **(A)** NRRL38499 and **(B)** NRRL54003 cultured on cellophane membrane overlaid on PDA were co-cultivated with four *Trichoderma* spp. and un-inoculated PDA (Control) for 40 h. After removing the cellophane membrane with *F. oxysporum* culture, one culture plug of *T. virens* strain G-41 was inoculated on each plate. Colony diameters after 2 days of incubation are shown. Values shown correspond to the mean ± SD of data from three replicates. Different letters indicate significant differences based on Fisher's test at *P* = 0.05.

To investigate whether the ability to respond to VCs from other fungi is unique to some *Trichoderma* spp., we evaluated the response of *F. oxysporum* strains NRRL38499 and NRRL54003 to VCs produced by four *Trichoderma* spp. (Figure 8). After exposing individual *F. oxysporum* strains cultured on sterilized cellophane membrane overlaid on PDA to *Trichoderma* VCs, we used *T. virens* to measure how strongly metabolites secreted by *F. oxysporum* inhibit its growth. VCs from all four *Trichoderma* spp. increased the amount of antifungal metabolites secreted by *F. oxysporum*. VCs from *T. viride* induced most in NRRL38499 (Figure 8A). However, for NRRL54003, VCs from *T. virens, T. harzianum*, and *T. viride* induced similarly, and the degree of induction by VCs from *T. asperellum* was significantly lower than the others (Figure 8B).

## Discussion

Microbes have evolved multiple mechanisms to interact with other organisms. The primary mechanism is the secretion of a wide variety of molecules (Braga et al., 2016; Vincent et al., 2016). Compared to rapid advances in research on how secreted proteins contribute to pathogenesis and symbiosis, research on secreted metabolites, especially those that are volatile, lags considerably behind in part due to an intrinsic bias caused by experimental design. Because studies on microbial interaction with other organisms typically rely on applying microbial cells, physical contact or waterborne molecules are often assumed to cause most responses. Using the assays described in Figure 1, we confirmed the role of VCs as a chemical weapon in *Trichoderma*-*F. oxysporum* interactions and also found that both fungi seem to recognize some VCs as an alarm for looming battle. We discuss how both types of VCs potentially contribute to biocontrol and fungal ecology below.

### Fungal VCs as a chemical weapon

Analysis of VCs produced by *T. virens* and *T. harzianum* (Table 3) revealed a few candidate compounds. Both species produced 3-octanone and 1-octen-3-ol, which are fungistatic and fungicidal (Wheatley et al., 1997; Okull et al., 2003). Two compounds produced by *T. harzianum*, 2-phenylethyl alcohol and isopentyl acetate, also exhibited antimicrobial activities (Ando et al., 2015; Medina-Romero et al., 2017). However, *T. virens* did not produce them. Compared to *T. harzianum, T. virens* produced more volatile sesquiterpenes (Figure 2). Lee et al. (2016) also reported that *T. virens* produced more sesquiterpenes than *T. harzianum*. It remains to be determined if any of these sesquiterpenes inhibit *F. oxysporum*, but some volatile sesquiterpenes have been shown to function as antimicrobials or plant growth regulators (Stoppacher et al., 2010; Yamagiwa et al., 2011; Ditengou et al., 2015; Kottb et al., 2015). Previous studies showed the production of 6-pentyl-2H-pyran-2-one, a secondary metabolite inhibitory to several plant pathogenic fungi, by *T. virens, T. viride, T. harzianum, T. atroviride*, and *T. koningii* (Scarselletti and Faull, 1994; Worasatit et al., 1994). However, we did not detect this compound in VCs produced by *T. harzianum* and *T. virens*, perhaps because its production may be strain specific or affected by the medium used, pH, light, water content, the age of culture, or combinations of these environmental factors (Lee et al., 2015).

The ability to use VCs as a weapon is not unique to *Trichoderma* as shown in Figure 1. Some antifungal compounds produced by *Trichoderma* spp., such as 2-phenylethyl alcohol and isopentyl acetate, were also produced by seven *F. oxysporum* strains (Table 5). Although none of the *F. oxysporum* strains appeared to produce 3-octanone and 1-octen-3-ol, we detected their production by NRRL38499 and NRRL26379 in our earlier study (Bitas et al., 2015), again suggesting the significance of environmental factors in determining which VCs are produced. Employment of VCs as a chemical weapon from both sides of interaction is not too surprising because diverse bacteria and fungi have been shown to produce antimicrobial VCs (Bitas et al., 2013; Bennett and Inamdar, 2015; Tyc et al., 2017). For example, VCs produced by the endophytic fungus *Muscodor crispans* strongly inhibited *Pythium ultimum, Phytophthora cinnamomi, Sclerotinia sclerotiorum*, and *Xanthomonas axonopodis* (Mitchell et al., 2010). A critical question is whether *Trichoderma* VCs affect the structure and activity of neighboring microbial communities in ways that impact the outcome of biocontrol. For example, suppression of beneficial plant-associated microbes may weaken the plant's ability to manage other stresses, causing reduced plant fitness. A related question is whether pathogens like *F. oxysporum* utilize antimicrobial VCs to manipulate plant-associated microbial communities to ensure its rhizosphere competency and pathogenicity. Identification of antimicrobial VCs and the genes involved in their synthesis will help answer these questions by allowing genetic manipulations of the synthesis of specific candidate VCs.

### Is VC-mediated recognition of *F. oxysporum* by *Trichoderma* a novel mechanism critical for biocontrol?

*Trichoderma* constitutively secretes a battery of enzymes, including proteases and cell wall degrading enzymes, at low levels (Kredics et al., 2003; Druzhinina et al., 2011; Vos et al., 2015). It has been hypothesized that specific peptides and cell wall components, released by the action of these enzymes, presumably function as signals for inducing the production of more enzymes and other weapons needed for mycoparasitism and directing growth (Woo et al., 2006; Seidl et al., 2009; Omann and Zeilinger, 2010; Druzhinina et al., 2011; Mukherjee et al., 2012). Consistent with this hypothesis, expression of *T. harzianum* genes encoding endochitinase CHIT42 and proteases PRA1 and PRB1 was highly induced in the presence of chitin (Samolski et al., 2009). Increased secretion of antifungal metabolites by *T. virens* and *T. viride* in response to *F. oxysporum* VCs (Table 4) suggests that both *Trichoderma* spp. sense *F. oxysporum* VC(s) to initiate preparation for pending attack. However, considering that the degree of VC-mediated growth inhibition by *Trichoderma* did not significantly increase after exposure to *F. oxysporum* VCs (Supplementary Figure 2), the induced antifungal metabolite(s) may not be volatile. Because the cellophane membrane used did not appear permeable to secreted proteins (Supplementary Figure 3), it remains to be determined whether *F. oxysporum* VCs also induce the secretion of antifungal proteins. The gene expression pattern in response to *F. oxysporum* VCs (Figure 7) suggests that the production of some such proteins might be affected.

In contrast to *T. virens* and *T. viride*, VCs from all *F. oxysporum* strains failed to induce the amount of antifungal metabolites secreted by *T. asperellum* (Table 4), and two *T. harzianum* strains responded to VCs from only some strains (Table 4 and Figure 6). However, the amount of unknown yellow metabolite secreted by *T. harzianum* (Figure 5) in response to *F. oxysporum* VCs suggested that VCs from some strains that did not induce the amount of antifungal metabolites but still affected *T. harzianum*. For example, VCs of NRRL38272 suppressed the amount of yellow metabolite secreted, while VCs of NRRL36118, NRRL37611, and NRRL22519 increased its secretion. VCs of NRRL26029, a strain that induced the amount of antifungal metabolites secreted by *T. harzianum* (Table 4), suppressed its secretion. Although we did not identify this metabolite, it probably is one of the chromogenic secondary metabolites called anthraquinones. Several *Trichoderma* spp., including *T. harzianum*, produce anthraquinones such as pachybasin, emodin, and ω- hydroxypachybasin (Reino et al., 2007). Some anthraquinones displayed antimicrobial activities (Chukwujekwu et al., 2006; Wu et al., 2006; Liu et al., 2009). However, it is not clear if its production is part of the preparation for chemical warfare because the amount secreted did not correlate with the degree of antifungal activity of secreted metabolites in response to *F. oxysporum* VCs (Table 4).

Differential expression of several biocontrol-associated genes in response to *F. oxysporum* VCs (Figure 7) also supports the hypothesis that *F. oxysporum* VCs affect cellular processes other than the secretion of antifungal metabolites in *Trichoderma*. However, similar to the secreted yellow metabolite, gene expression pattern did not present a clear picture of how VCs of individual strains affected *Trichoderma*. Although VCs of NRRL38487 and NRRL37616 induced the amount/activity of antifungal metabolites secreted by *T. virens* and *T. harzianum* much more strongly than VCs of NRRL26379 and NRRL38499, VCs of the former two significantly induced the expression of only *prb* (subtilisin-like protease) and *hsp* (small heat-shock protein) in *T. virens*. As for *chit* (chitinase), VCs of the latter two significantly induced its expression. All three genes were reported to be up-regulated by contact with pathogens (Samolski et al., 2009; Seidl et al., 2009; Vieira et al., 2013). In *T. harzianum*, all three genes appeared to be suppressed or unaffected by *F. oxysporum* VCs. Expression of *qid*, a gene that encodes a cell wall protein presumably involved in protecting cell wall from degradation (Rosado et al., 2007; Vieira et al., 2013), was induced in *T. virens* by VCs of NRRL38499 (mild inducer of the amount of antifungal metabolites secreted) and NRRL38487 (strong inducer), but not by VCs of the other two. Only VCs from NRRL38499 significantly induced *tps*, which encodes a terpene synthase, in *T. virens*. Most of the *T. harzianum* genes were down-regulated or unaffected by *F. oxysporum* VCs (Figure 7).

We hypothesize that pathogen recognition by sensing pathogen-derived volatile cues helps biocontrol. Complex molecular responses of *Trichoderma* spp. to *F. oxysporum* VCs (Table 4 and Figures 4–7) indicated several experiments needed to test this hypothesis. Global gene expression analysis of more *Trichoderma* strains/species via RNAseq will help better understand how *F. oxysporum* VCs affect *Trichoderma* BCAs and if any resulting changes are related to biocontrol. Another important work is determining how other *Trichoderma* spp. respond to VCs from diverse pathogens. Considering the phylogenetic positions of the four species tested (Kubicek et al., 2003, 2011), this ability does not appear to follow their evolutionary relationships. Perhaps, the most critical question is which VC(s) function as signals and how *Trichoderma* recognizes and processes such signals (e.g., receptor-mediated or other ways?). Analysis of VCs produced by seven *F. oxysporum* strains (Table 5) revealed that although each strain produced a unique mix of VCs, all of them produced six compounds. One of the compounds, 2-phenylethyl alcohol, was reported to stimulate the developmental transition of *Saccharomyces cerevisiae* from unicellular to filamentous (Chen and Fink, 2006). Similarly, 3-octanone and 1-octen-3-ol also regulate fungal development and mediate inter-colony communication in *Penicillium* and *Trichoderma* (Chitarra et al., 2004; Nemcovic et al., 2008). Real-time analysis of VCs using GC-MS with improved sensitivity would provide more accurate information about the composition and amount of candidate VCs produced by *F. oxysporum* over time.

### Additional questions and future directions

Recognition of *Trichoderma* VCs by *F. oxysporum* and vice versa (Table 4 and Figure 8) suggests that this form of interaction may occur between other fungi. As noted in Introduction, such VC-mediated interactions seem to occur even between bacteria and fungi (Spraker et al., 2014; Rybakova et al., 2017), which underscores the need to survey many combinations of microbes to study how such manipulations influence the structure and activity of microbial communities.

The methods we employed (Figure 1) can support rapid screening of diverse combinations of microbes to study if they interact via VCs. However, there exist some limitations. Although cellophane membrane allowed us to quantify the amount of antifungal metabolites secreted and harvest mycelia easily for subsequent analysis, it is not suitable for evaluating fungal strains that secrete enzymes with high cellulolytic activity. We observed that hyphae of some strains reached medium by breaching the membrane. Analysis of secreted metabolites is needed to identify antifungal compound(s) and how VCs control their synthesis. However, their presence in solid medium makes this analysis challenging. As noted above, changes in the secretion of antifungal proteins in response to VCs could not be determined. Considering that VCs from both sides likely affect each other in multiple ways, an assay system that allows unidirectional airflow would be highly desirable to determine how VCs from one culture affect the other.

## Author contributions

NL, AA, and WW performed most of the experiments and wrote the manuscript. MI and KN performed some of the experiments. XL contributed to GC-MS analyses. SK coordinated the study, designed experiments, and revised the manuscript.

### Conflict of interest statement

The authors declare that the research was conducted in the absence of any commercial or financial relationships that could be construed as a potential conflict of interest.
