# Supplementary material for: Volatile Compound-Mediated Recognition and Inhibition Between Trichoderma Biocontrol Agents and Fusarium oxysporum
Source: Front Microbiol. 2018 Oct 31;9:2614. doi: 10.3389/fmicb.2018.02614 (PMC6231246; doi:10.3389/fmicb.2018.02614)
Supplement: Supplementary file 1 [file Table_1.DOCX]

**Supplementary Table 1.** **Primers used for qPCR analysis**

| **Gene** | **Transcript ID** | **Size (bp)**^a^ | **Forward primer (5’-3’)** | **Reverse primer (5’-3’)** |
| --- | --- | --- | --- | --- |
| ***T. virens*** | | | | |
| *chit* | 189743 | 105 | TTGCTCGCTCTTGTTCCTTC | TGCTTTGTGAGTTGGCTGAG |
| *endo* | 218832 | 131 | AGCTGGCAAGGACAACTACT | CGAACAGATTGGCGTCATGT |
| *bgn* | 231316 | 136 | ACAAGCTCAACCACGCATAC | GGCATGTCGTCCTTGTTGTT |
| *pra* | 181449 | 102 | GGCACTTGTCAACCCCAATT | CTGCGACGGTAAGAGTTGTG |
| *prb* | 186844 | 126 | CCTCGGTAATCCTTGACGGT | TAATAGCGGTAGTCCAGGCG |
| *qid* | 230780 | 145 | CAACAACAAGGGCGAAGTGT | AGCTTCCTTGCCACAGTCTA |
| *hsp* | 195080 | 111 | GTGAGCCTGGTGTTGGAAAG | CGACATCAAGCGAGAGAAGC |
| *acs* | 203203 | 120 | ATACATTTTCTCAGGCCGCG | GCAGGCTTCGGTCACATAAG |
| *ans* | 193889 | 116 | ACAGGTAGTGGGCATCTTCC | GCCCATTCAATCGGTTCCTC |
| *pks* | 204589 | 147 | TCTGCGATGAAAAGTGACCG | CCTTTCTAATGACCGCTGCC |
| *tps* | 141474 | 151 | TCAGACGGGCCTTGAATAAG | TCTTCTCCTCTGCGACATTG |
| *tef* | 216092 | 97 | GTTGCTTTCGTCCCCATCTC | CCTTGGTCTCCTTCTCCCAG |
| *act* | 229062 | 108 | TGGCACCACACCTTCTACAA | CTGGGTCATCTTCTCACGGT |
| ***T. harzianum*** | | | | |
| *chit* | 529621 | 103 | CAGCAGCGTCTCTCCTTCTA | TTGGCAAAGTTGGTCATGGG |
| *endo* | 101028 | 137 | CCTCAAGGTTCTGCTCTCCA | CAATGCCATCGAAACCCCAA |
| *bgn* | 485240 | 136 | ACAAGCTCAACCACGCATAC | GGCATGTCGTCCTTGTTGTT |
| *pra* | 526221 | 126 | CACTGCACCATTGACCAGAG | CGACAGTGTAGCTAGGGTGA |
| *prb* | 110777 | 140 | TATGATACCTCGGCTGGCAG | CCATGGCCAAGAGTGTCAAC |
| *qid* | 509414 | 81 | CTTAAGCAGGTCCTTGTGGC | CACGTCATGGAGGTTGTTCG |
| *hsp* | 8037 | 148 | CATCGAACGAGCACATGGAG | TTGCAAGTCTTGGGCTTTCC |
| *acs* | 479794 | 99 | TTCTTGGCCCTGGAGAAGAG | CAGACCACGACTTCTCCACT |
| *ans* | 534341 | 130 | ATCTCAAGACCTCACGCACA | GCCCATTCAATCGGTTCCTC |
| *pks* | 521432 | 125 | CGATGAAGAGTGACCGCAAG | CCTTTCTAATGACCGCTGCC |
| *tps* | 523651 | 122 | GTGCCAGATATTCTTGCGCT | TCTTCTCCTCTGCGACATTG |
| *tef* | 12328 | 97 | GTTGCTTTCGTCCCCATCTC | CCTTGGTCTCCTTCTCCCAG |
| *act* | 510028 | 90 | TGGCACCACACCTTCTACAA | GTTGGACTTGGGGTTGATGG |

^a^ Expected size of each amplicon is shown.

**
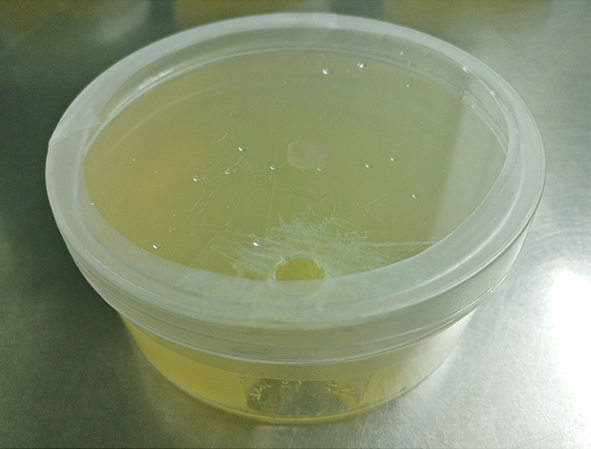
**

**Supplementary Figure 1. Culture dish used to collect *F. oxysporum* VCs.** 250 ml PDA was used for culturing *F. oxysporum.* One hole (5 mm in diameter) was created on the lid using a flamed cork corer and sealed using a piece of tape. A PDA block beneath the hole was removed before inoculating *F. oxysporum*.

**
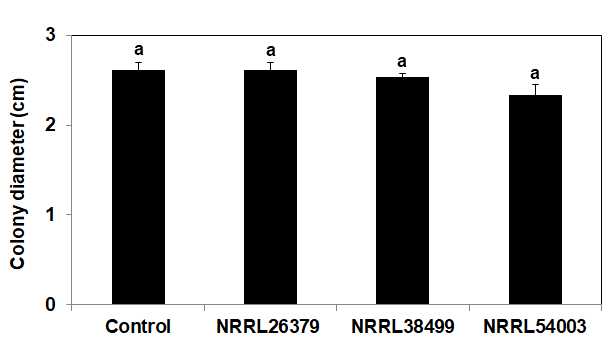
**

**Supplementary Figure 2. Effect of *F. oxysporum* VCs on the amount/activity of anti-fungal VCs produced by *T. virens*.** *T. virens* was co-cultivated with three *F. oxysporum* strains and un-inoculated PDA (Control) for 40 h as shown in **Figure 1**. After replacing each *F. oxysporum* plate with a plate inoculated with a plug of NRRL54003 culture, they were co-cultivated for 3 days. Colony diameters shown correspond to the mean ± SD of data from three replicates. Different letters indicate significant differences based on Fisher’s test at *P*=0.05.

**
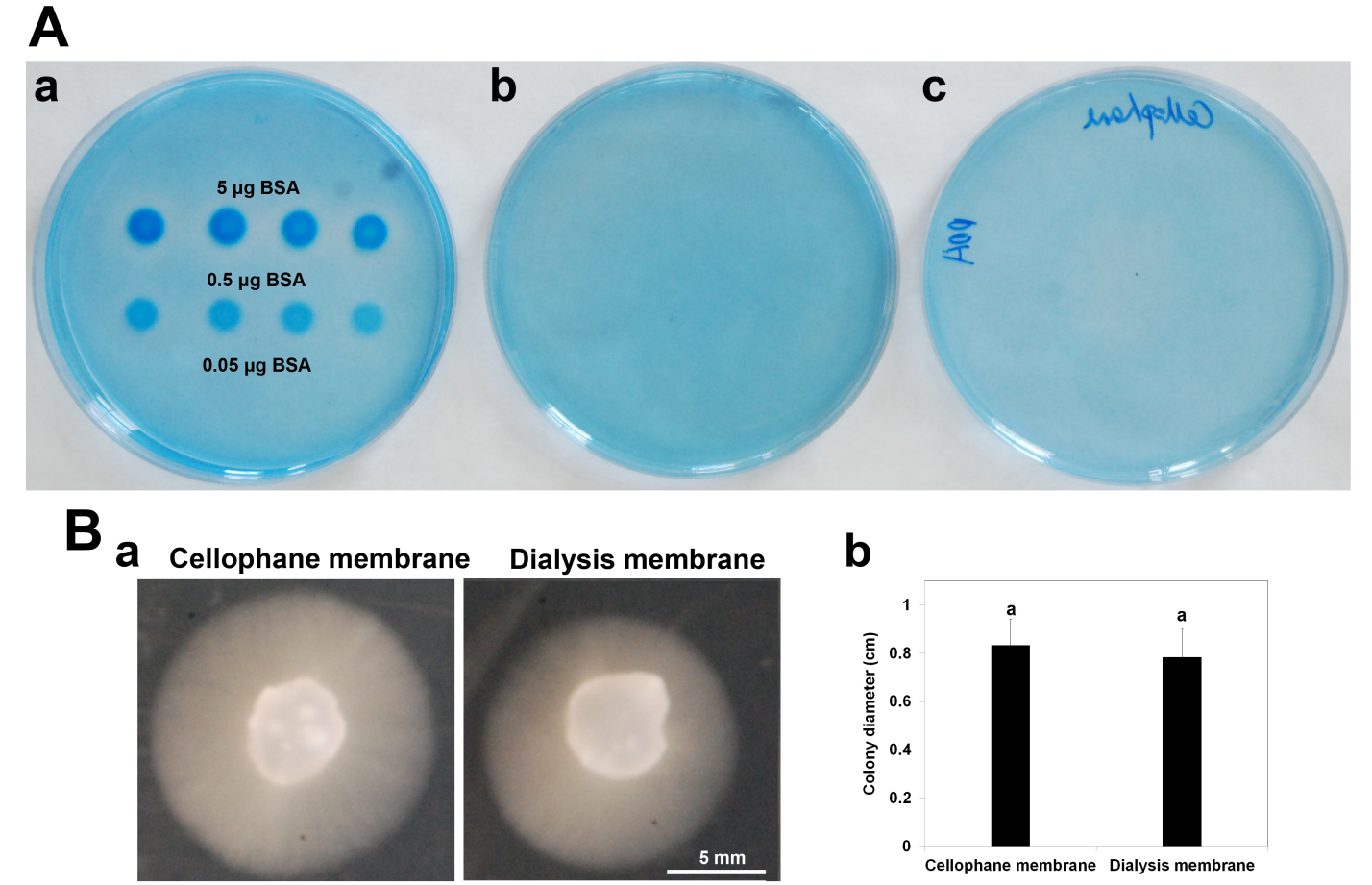
**

**Supplementary Figure 3. Evaluation of the protein permeability of cellophane membrane. A**, protein staining was performed after applying four drops of bovine serum albumin (BSA) solutions (5 μg, 0.5 μg and 0.05 μg of BAS per drop) on (**a**) PDA and (**b**) cellophane membrane overlaid on PDA. (**c**) After culturing *T. virens* strain G-41 on cellophane membrane overlaid on PDA for 40 h, cellophane membrane was removed. Photographs were taken after 1h of protein staining at room temperature. **B**, *T. virens* strain G-41 was inoculated on cellphone membrane (left) and dialysis membrane (right) overlaid on PDA and cultured for 40 h. After removing membrane, one plug of NRRL 54003 culture was inoculated on each plate. Colony **(a)** morphology and (**b**) diameter after 3 days of growth are shown. Values shown correspond to the mean ± SD of data from three replicates. No significant difference was detected based on Fisher’s test at *P*=0.05.
